# Conservative Management of a Scar Abscess formed in a Cesarean-induced Isthmocele

**DOI:** 10.3389/fsurg.2016.00007

**Published:** 2016-02-16

**Authors:** Meriem Boukrid, Jean Dubuisson

**Affiliations:** ^1^Department of Gynecology-Obstetrics, University Hospital of Geneva, Geneva, Switzerland

**Keywords:** scar abscess, cesarean-induced isthmocele, uterine discharge after cesarean

## Abstract

**Background:**

Abscesses located in the cesarean-section (CS) induced isthmoceles are rarely encountered and are usually treated surgically, mostly by hysterectomy.

**Case description:**

We here report the case of a 40-year-old primiparous woman presenting a symptomatic abscess in the isthmocele 10 years after a CS. She was treated by antibiotics and was closely monitored by clinical evaluation, ultrasonography, and pelvic magnetic resonance imaging. This treatment led to complete resolution of symptoms and a disappearance of the abscess at imagery.

**Conclusion:**

Our report shows that a conservative medical management of isthmocele abscesses can be an effective approach in women wishing to preserve their uterus.

## Introduction

The cesarean delivery rate is steadily increasing worldwide (1). Cesarean sections (CSs) and resulting uterine scars are associated with obstetric complications, such as cesarean scar pregnancies, uterine rupture, abnormal placental implantation, and secondary infertility (2). Surgical site infection after CS is rare and happens mostly within 30 days (3). The reported rate of abdominal wound infection under prophylactic antibiotic coverage is usually around 0.52% (4). We present a rare case of a CS scar abscess having developed 10 years after delivery that was successfully managed by conservative antibiotic therapy.

## Materials and Methods

A 40-year-old woman, primigravida, primiparous by CS, attended our hospital with a 3-week history of malodorous uterine discharge, and recent dysmenorrhea. She was not on any contraception. Her CS had taken place 10 years previously for non-progression and her baby had been born in good condition. There were no risk factors for sexually transmitted diseases. Physical examination revealed apyrexia with abdominal tenderness. Speculum inspection showed a brown malodorous cervical discharge with pus, and digital examination of the uterus was painful.

**Figure 1:**
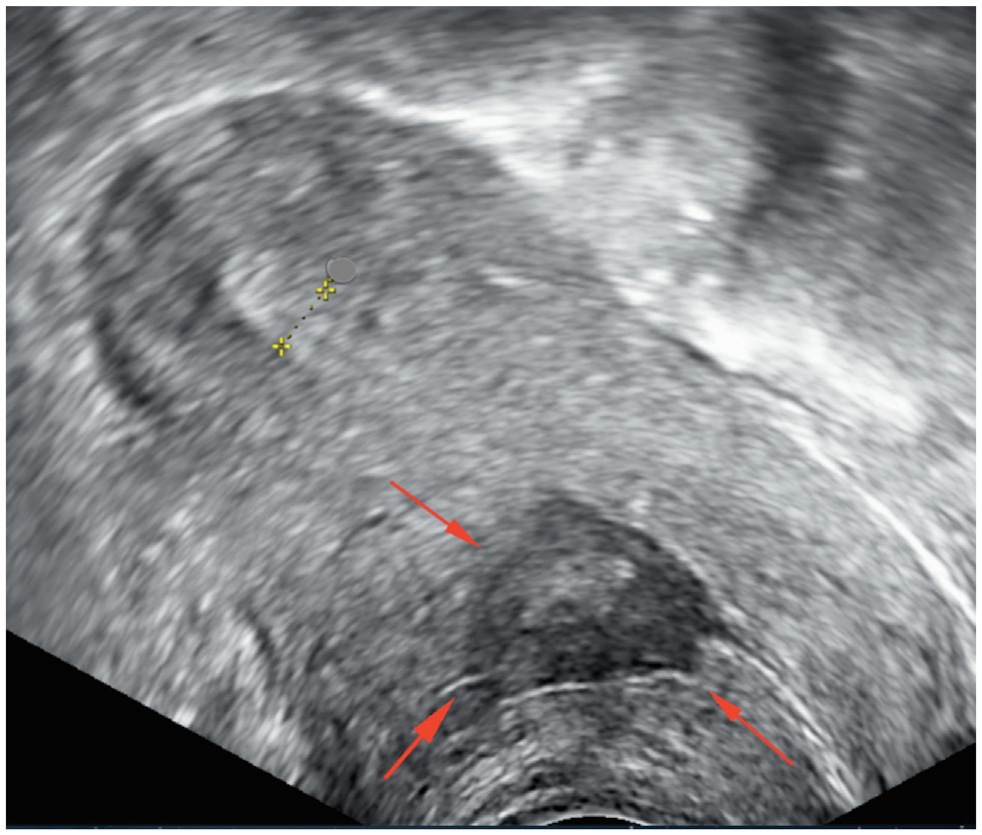
**Pelvic ultrasonography before treatment shows a heterogenic cavity at the site of cesarean section**.

Pelvic ultrasonography demonstrated an ante-flexed, normal-sized uterus with a spherical mass within the uterus scar tissue between the cervix and the body of uterus, involving myometrium and measuring 39 mm × 14 mm × 23 mm. The center contained mixed echogenic and anechogenic material, evoking liquid and tissue components. The myometrium covering the lesion was thickened. There was no sign of adenomyosis. The endometrium measured 6 mm and was of normal appearance. Both ovaries were normal and there was no intra-peritoneal fluid (Figure 1).

**Figure 2:**
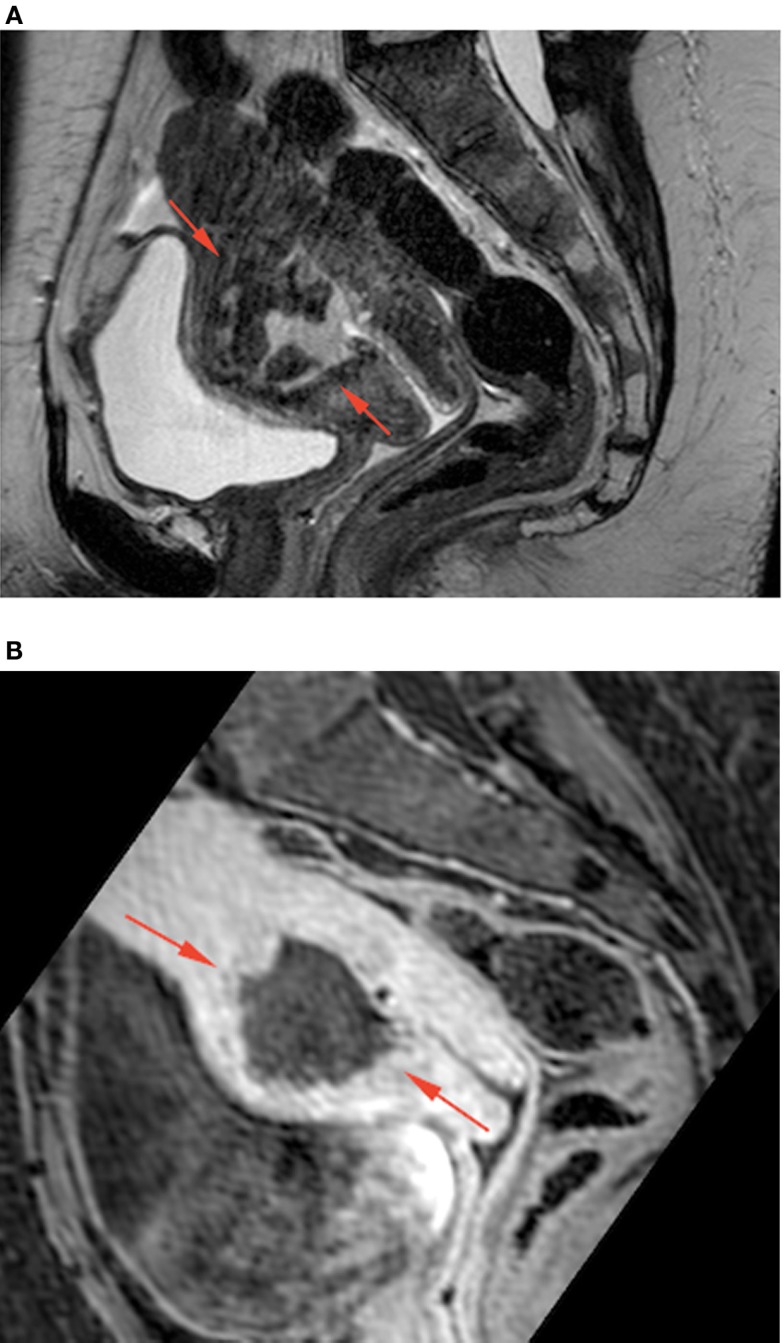
**Sagittal T2-weighted TSE (A) and Dixon T1-weighted fat saturated after iv gadolinium injection (B) MRI images at presentation, showing a cavity with thick enhancing walls (red arrows) containing heterogeneous fluid and solid non-enhancing material at the anterior wall of the uterus at the site of cesarian section**.

Inflammation markers were slightly raised and a pregnancy test was negative. Cervical bacteriology was not contributive. Endometrial biopsy revealed purulent material with a lot of leukocytes and rare fragments of benign endometrial epithelium. We suspected an abscess. Magnetic resonance imaging (MRI) showed a well-defined uterine mass embedded in myometrium, made-up of mixed liquid and tissue components, without hypersignal in diffusion rating and taking up contrast peripherally (Figure 2).

These images lead to a differential diagnosis, including necrotic fibroid and myosarcoma. Diagnostic hysteroscopy was difficult; we found a lot of inflammatory material, pus, and revealed necrotic tissue located in the lower anterior wall, forming a new cavity (“isthmocele”). We found no blood inside the cavity. The rest of the endometrial cavity was of normal appearance. All these elements led us to suspect an infection. Hence, a scar abscess having developed in a cesarean-induced isthmocele was suspected. We decided to attempt conservative treatment. She was a patient with an unremarkable medical history wishing to preserve her fertility. She was empirically treated with amoxicillin–clavulanic acid 1 g/12 h for 2 weeks with good tolerance.

## Results

**Figure 3:**
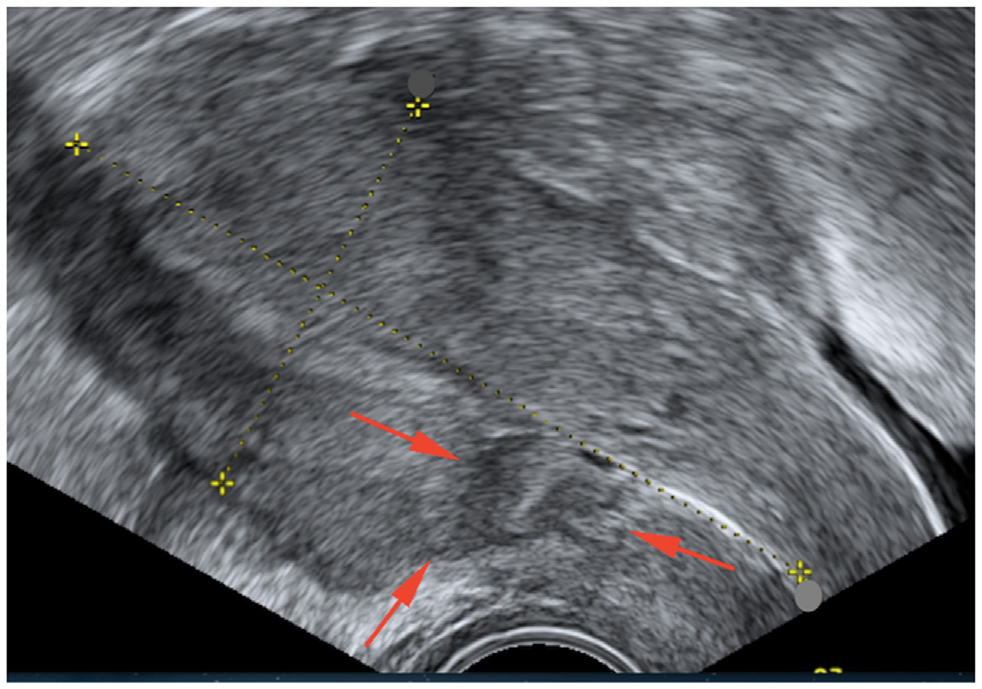
**Pelvic ultrasonography after medical treatment shows a small residual heterogenic cavity at the site of cesarean section**.

At the end of the 2 weeks, the patient was no longer symptomatic and a repeat ultrasound showed a reduction of the lesion of about 50% to 23 mm × 12 mm (Figure 3).

**Figure 4:**
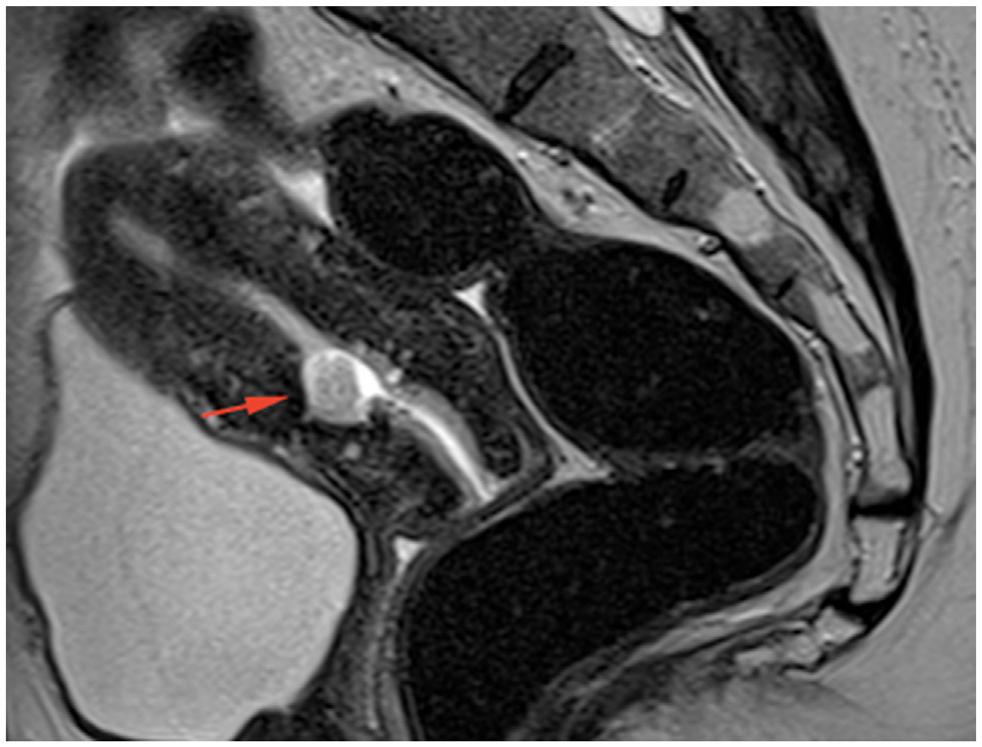
**Sagittal T2-weighted TSE MRI image 1 month after medical treatment showing a small residual cavity containing some debris at the site of the abscess**.

MRI 1 month later showed the disappearance of the entire lesion and the residual myometrial scar measured 5 mm (Figure 4).

The patient remained asymptomatic at 20 months follow-up. The clinical response to the antibiotics and the results of imaging confirm our suspected initial diagnosis.

## Discussion

New complications are appearing with the recorded increase in number of CS. The relative risk reduction with prophylactic antibiotics in maternal infection is well known, the incidence currently hovering around 2% for post-partum endometritis and around 0.52% for superficial wound infection (5). Infection after CS usually occurs within the first 30 days after delivery (3). Barbut et al. identified primiparity, CS in emergency circumstances or during labor and post-partum bacteriuria as the main risk factors for infective complications. Microorganisms most frequently identified include enterobacteria, anaerobes, *staphylococcus aureus*, and *enterococcus*. An abscess developing at the uterine incision site long after a CS is considered very rare. Only two cases have so far been reported (shown in Table 1 with their characteristics). One 6 years after CS treated by hysteroscopic and laparoscopic surgery after failed antibiotics (6), and one 8 years after CS treated by hysterectomy (7). Defects have been described in the scar location after CS in 4.8% (8, 9) such as myometrial discontinuity at the site of a previous CS scar, including the production of mucus, blood, and the collection of menstrual product (10–12). Abnormal uterine bleeding and lower abdominal pain are observed in 46% of women with cesarean scar dehiscence (10). The risk increases with the number of CS and with a retroflexed uterus. The collection may get infected locally which would explain the finding of leukocytes and signs of necrosis in the endometrial biopsy. Ultrasonography, CT, and MRI are useful tools in evaluating the location, dimension, and etiology of a pelvic abscess (13). To this day, the only cases reported were managed surgically, mostly by hysterectomy. In our case, as the patient wished to preserve her fertility, we opted for conservative management with amoxicillin–clavulanic acid covering a wide selection of gynecological infections. We were successful and complete resolution of the abscess was achieved.

**Table 1:** **Summary of reported cases of cesarean abscess**.

Author characteristics	*Diaz-Garcia* (6)	*Takako Taguchi* (7)	*Present case*
Year	2009	2012	2015
Age (years)	36	44	40
Number of CS	1	1	1
Time after CS (years)	6	8	10
Symptoms	Fever, abdominal pain	Fever, abdominal pain, abnormal uterine bleeding	Abnormal uterine bleeding, dyspareunia
Size of abscess (cm)	2.4 × 3 × 1.9	12 × 10 × 10	3.9 × 1.4 × 2.3
Treatment	Antibiotics followed by laparoscopic and hysteroscopic reconstruction	Antibiotics followed by total abdominal hysterectomy	Antibiotics

Close monitoring with ultrasound and MRI allowed us to continue managing this patient conservatively.

## Conclusion

Cesarean section scar abscess after 10 years is a very rare complication. Symptoms seem to be related to the CS defect. To our knowledge, this is the first case describing successful conservative management leading to the complete resolution of symptoms and disappearance of radiological evidence. In our patient, pus and necrosis on endometrial biopsy oriented us toward local infection rather than a malignant process and the patient was systemically well and symptoms improved with treatment. This case shows that under these circumstances, surgery may be kept as a second-line option or can even be completely avoided.

## Ethics Statement

Written informed consent was obtained from the patient prior to presenting the case.

## Author Contributions

All authors listed, have made substantial, direct and intellectual contribution to the work, and approved it for publication.

## Conflict of Interest Statement

The authors declare that the research was conducted in the absence of any commercial or financial relationships that could be construed as a potential conflict of interest.
